# Newly optimized ELISA kit and LAT reveal significantly higher seroprevalence in sheep raised in agro-ecological zone as against range-ecological zone, with a significant association of meteorological parameters

**DOI:** 10.1371/journal.pone.0290374

**Published:** 2023-12-05

**Authors:** Sarfraz ur Rahman, Haroon Akbar, Muhammad Zubair Shabbir, Ubaid Ullah, Muhammad Imran Rashid

**Affiliations:** 1 Faculty of Veterinary Science, Department of Parasitology, Molecular Parasitology Laboratory, University of Veterinary and Animal Sciences, Lahore, Pakistan; 2 Institute of Microbiology, University of Veterinary and Animal Sciences, Lahore, Pakistan; Institute of Tropical Medicine: Instituut voor Tropische Geneeskunde, BELGIUM

## Abstract

**Background:**

*Toxoplasma gondii* is a zoonotic and foodborne intracellular parasite capable of inducing congenital infections, stillbirths and abortions in humans and animals, globally. The consumption of undercooked or raw mutton is “one of the vital risks” for acquiring toxoplasmosis: an asymptomatic condition in healthy persons, while life-threatening in immunodeficient individuals like "HIV/AIDS" patients.

**Objectives:**

The current study has multiple objectives: to optimize a newly ELISA kit for Sheep, to find out the seroprevalence of ovine toxoplasmosis of two ecological zones of the Punjab, Pakistan through LAT and newly Optimized Sheep ELISA kit, to do the comparison of efficacies of various tests (LAT with newly Optimized ELISA kit and newly Optimized ELISA kit with commercial ELISA kit) and to determine the different meteorological parameters as the risk factors for *T*. *gondii* infection in sheep.

**Methods:**

A cross-sectional study was conducted on 400 sheep sera, 200 were collected from sheep raised on open grazing system by local farmers in the adjoining areas of Civil Veterinary Dispensaries (CVDs) of range-ecological zone i.e. tehsil Kot Chutta (Dera Ghazi khan). Similarly, the remaining 200 were collected from agro-ecological zone i.e. tehsil Sharaqpur (Sheikhupura), to evaluate the comparative efficacy of LAT with optimized ELISA kit and newly optimized ELISA kit with commercial ELISA kit.

**Findings:**

The newly ELISA kit optimized against a commercial ELISA kit was found to have 100% sensitivity, 97.6% specificity with 98% Positive Predictive Value, 100% Negative Predictive Value, Cut off value = 0.505, 28.28 LR+, 0.0104 LR-, and 2719.23 DOR. Seroprevalence of toxoplasmosis was detected significantly (P < 0.01; χ2) higher in Sharaqpur (44.5% by LAT; 35.5% by ELISA) as compared to that in Kot Chutta (39.5% by LAT; 31% by ELISA). The highest seroprevalence was seen in the sheep of the 1–2 years age group (P < 0.01; χ2), whereas the lowest in the oldest animals (≥ 4 years). Investigation of meteorological data of both the regions reveals that the zone with higher seroprevalence has relatively higher rainfall, higher humidity, lower environmental temperatures, and higher altitude as the critical factors, potentially behind the significant difference seen in seroprevalence level. The partial correlation of both tests (newly optimized ELISA kit and LAT) was 0.991 at maximum temperature in Sharaqpur while it was 0.981 in Kot Chutta.

**Interpretation:**

A novel significant correlation was found between the meteorological parameters (relative humidity, minimum, maximum, and average temperatures) divided into yearly units of both the ecological zones, and year-wise seroprevalence (birth years of age-wise groups) of the corresponding regions. We hypothesize that such environmental conditions increase the risk of toxoplasmosis in grazing sheep, owing to a more favorable environment for coccidian oocyst survival. The ELISA kit optimized in this study will be helpful for the detection of seroprevalence of ovine toxoplasmosis in other ecological zones of Pakistan as well as of any other country in the world. More studies are recommended involving regions from other ecological zones of Pakistan to further explore the seroprevalence of ovine toxoplasmosis and to ratify the novel correlation of meteorological parameters with seroprevalence.

## Introduction

*Toxoplasma gondii* can be transmitted by consumption of raw or undercooked meat [1]. *T*. *gondii* is a cosmopolitan parasite infecting warm-blooded vertebrates across the planet, including humans, animals, and birds [2]. The adverse outcomes of this disease seen in sheep and goats include fetal resorption, congenital anomaly, and abortion, thus leading to economic losses [3]. Additional economic losses due to toxoplasmosis, also occur through a reduction in the number of lamb births, a reduction in milk production, and an increased incidence of post-abortion complications such as delayed fertilization and vaginal infection [4]. Humans usually get toxoplasmosis by eating barbecue or raw meat, particularly mutton of sheep origin having bradyzoites of *T*. *gondii* [5,6]. A highly diverse prevalence of toxoplasmosis has been reported in sheep around the world, ranging from 28.5% to 78% [7]. Seroprevalence of toxoplasmosis in humans (65% to 71%) is higher than that seen in many other mammalian hosts like 58.57% in rats, 25% in cattle, 52% in goats, and 24% in sheep in Pakistan [8].

Various recombinant proteins of *T*. *gondii* are employed for the diagnosis of anti-*T*. *gondii* antibodies in animals are mostly surface antigens (SAG) [9–14], dense granules (GRA) [14–18], micronemes (MIC) [19–21], matrix [22] and rhoptry (ROP) [14,23] proteins of *T*. *gondii*. Various studies have demonstrated that recombinant antigens are highly effective in detecting toxoplasmosis for diagnostic purposes. A recombinant antigen or combination of different antigens can be employed to enhance sensitivity for the detection of anti-*T*. *gondii* antibodies [21].

Immunological assays constitute an excellent tool to assess farm infection levels [24,25]. Various techniques exploited for surveillance in live animals are comparatively accurate, low-cost, and need a minute quantity of the sample [26–28]. Various serological tests like Dye Test (DT), Immunofluorescence Antibody Test (IFAT), Modified Agglutination Test (MAT), and Enzyme-Linked Immunosorbent Assay (ELISA) have been employed for the serodiagnosis of toxoplasmosis.

The use of a low-cost preliminary screening test like Latex Agglutination Test (LAT) is a good option for surveillance studies, whereas some tests are not only costly but carry a difficulty of interpretation, like the Dye Test [29,30]. On the other hand, ELISA is considered a preferred serological technique with higher sensitivity and specificity for screening large numbers of samples for toxoplasmosis [29,31].

ELISA is a more sensitive and more specific technique for detection of antibodies against *T*. *gondii* in contrast to Modified Agglutination Test (MAT) in sheep and other host species like dogs and cats [32], hence a great tool to be exploited for seroprevalence studies for this zoonotic disease of One-Health importance. In a previous study, SAG1 was used in an ELISA-based diagnosis of specific antibodies against *T*. *gondii* in a mouse model [33], leading to the development of Toxo ELISA kit to diagnose toxoplasmosis in human beings [34]. Out of various recombinant proteins, i.e., surface-antigen-1 is considered an excellent candidate for use in serodiagnostic kits for serodiagnosis of *T*. *gondii*-specific antibodies, i.e., seroprevalence studies [35,36]. This is due to the fact that surface-antigen-1 (SAG1) is an immunodominant surface antigen of *T*. *gondii* and it is helpful for identification of antibodies against *T*. *gondii* [37–39]. Recombinant protein-based ELISA kits can also be exploited to identify current and past episodes of toxoplasmosis [40,41] by adapting the kit to detect IgM and IgG, respectively. This study has been designed for multi-purposes: to optimize a new ELISA kit (named as Sheep Toxo IgG ELISA kit) and to assess the seroprevalence of ovine toxoplasmosis of range-ecological zone and agro-ecological zone of the Punjab, Pakistan by LAT and Sheep Toxo IgG ELISA kit. The evaluation of tests characteristics (LAT with newly Optimized ELISA kit and newly Optimized ELISA kit with commercial ELISA kit) was done and analysis of different meteorological parameters as the risk factors for *T*. *gondii* infection in sheep was also performed.

## Methods

### Ethics statement

In this study, we strictly followed the recommendations of the Guide for the Care and Use of Animals of the University of Veterinary and Animal Sciences (UVAS), Lahore, Pakistan. The protocol was approved by the Animal Ethics Committee of UVAS, Lahore, Pakistan (permission number DR/956, dated: 19-09-2019). All the procedures of blood collection and the experimental protocols were implemented under the efforts to alleviate suffering.

### Study design

The newly ELISA kit for sheep was optimized through the evaluation of 90 sera which were selected from 400 that were collected from the areas of Civil Veterinary Dispensaries (CVDs) of Kot Chutta and Sharaqpur. Ovine seroprevalence of Kot Chutta and Sharaqpur was determined by LAT and newly optimized ELISA kit. The comparison of efficacies of various tests (LAT with newly optimized ELISA kit and commercial ELISA kit with optimized ELISA kit) were determined through finding out the sensitivity, specificity, LR+, LR-, DOR and ROC curve and Kappa value using MedCalc (version 11.4.4.0) software. Subsequently, meteorological parameters as risk factors were analyzed using SPSS (IBM SPSS Statistics for Windows, Version 23.0. Armonk, NY: IBM Corp) and R-4.2.1 software.

### Collection of sheep sera

The blood samples were collected from the Juglar vein of sheep by 3ml disposable syringes and efforts were done to alleviate suffering of sheep. These collected bloods were poured into yellow cap blood collection vacutainers. These were brought to the Molecular Laboratory of Parasitology, University of Veterinary and Animal Sciences, Lahore, Pakistan. A total of 400 sheep sera were collected while recording the age as well as the sex of the animals. Sheep were of Lohi and Kajli breeds. Out of 400, 200 sera were collected from sheep raised on open grazing system by local farmers in the adjoining areas of Civil Veterinary Dispensaries (CVDs) of range-ecological zone i.e. tehsil Kot Chutta (Dera Ghazi khan). All the animals were healthy males (*n* = 82) and females (*n* = 118), belonging to various age groups: ≤ 1 year (*n* = 27), 1–2 years (*n* = 101), 2–3 years (*n* = 34), 3–4 years (*n* = 33) and ≥4 years (*n* = 05).

Similarly, the remaining 200 sera were collected from agro-ecological zone i.e. tehsil Sharaqpur (Sheikhupura). All the animals were healthy males (*n* = 74) and females (*n* = 126), belonging to various age groups: ≤ 1 year (*n* = 40), 1–2 years (*n* = 85), 2–3 years (*n* = 38), 3–4 years (*n* = 27) and ≥4 years (*n* = 10).

### Newly optimized ELISA kit

Out of 400, ninety (90) sera of sheep were processed to optimize our new ELISA kit against a commercial ELISA kit (ID. vet Diagnostics, France, Cat # TOXOS-MS-2P). A commercial ELISA kit was employed as described by the manufacturer’s protocol (ID. vet Company). Briefly, sera were dispensed into wells of 96-well plate with 1:10 dilutions, respectively. One well was put as blank, whereas two wells were for negative control and two for positive control. The plate was put into an incubator at 26°C for 30min and washed with a washing solution three times. 100μL of Anti-multi-species IgG-HRP conjugate was added, incubated at 26°C for 30min, and washed on the plate three times. Subsequently, 100μL Tetramethylbenzidine (TMB) substrate was added, incubated (26°C/15min), and added 100-μL of stop solution (1N HCl). After that, OD was recorded at 450nm by an ELISA reader (ELX-800, BioTek, USA). The following formula decides the fate of a sample as a positive or a negative: Sample-to-positive (S/P)% = {(Optical Density Sample-Optical Density NC)/(Optical Density PC-Optical Density NC)}*100. If (S/P)% was ≤ forty (40), the sample is believed as negative; if (S/P)% was between forty (40) and fifty (50), the sample is believed as ambivalent, and in case (S/P)% was ≥ fifty (50), the sample is considered as positive.

The protocol of our ELISA kit is different from that of the Commercial ELISA kit. Briefly, rSAG1 was coated on the wells of 96-well plate (JET BioFil, China) @ 0.125μg/mL in 50mM Na_2_CO_3_ buffer followed by overnight incubation (4°C). It was washed (5 times) with 1X Phosphate Buffered Saline/0.05% Tween-20 @ 300μL/well. The blocking was done with 4% Bovine Serum Albumin /1X PBS (200μL/well), an incubation step (37°C/2hrs.), and then washing was done (5 times) with the washing solution. Ninety sera were disbursed into wells with dilution at 1:1600. The negative and positive control sera were added in two wells. The positive and negative controls were taken from standard ELISA kit (ID Screen Toxoplasmosis Indirect Multi-species, ID. vet, Grabels, France). For blank, two wells were set in the plate. The coated plate was incubated (37°C/2hrs.), and washing was done (5 times). Donkey anti-sheep IgG (H+L) antibodies-AP (Invitrogen, Thermo Fisher Scientific, Cat # A16044) were disbursed at @1:10,000 dilution (100μL/well), and a 96-well plate was incubated (37°C/2hrs.) and then washing was performed (5 times). P-nitrophenyl phosphate (Thermo Scientific, USA, Ref # 34045) was prepared through 1mg/mL of Diethanolamine, and the prepared substrate was dispensed (100μL/well). Incubation of plate was done at 37°C, and reaction was stopped with 1M NaOH at 100μL/well within fifteen min. OD was taken at 405nm through an ELISA reader for a 96-well plate (ELX-800, BioTek, United States of America). The criteria for deciding between positive and negative samples tested in an optimized ELISA kit were as follows: any sample having 2.5-fold the mean of OD values obtained by two negative controls was regarded as a positive sample. The following formula decides the fate of a sample as a positive or a negative: Sample-to-positive (S/P)% = {(Optical Density Sample-Optical Density NC)/(Optical Density PC-Optical Density NC)}*100. If (S/P)% was ≤ forty (40), the sample is believed as negative; if (S/P)% was between forty (40) and fifty (50), the sample is believed as ambivalent, and in case (S/P)% was ≥ fifty (50), the sample is considered as positive.

### Determination of cut off, accuracy, kappa value, ROC, and area under curve

OD values gained in our optimized ELISA and in the Commercial ELISA were applied in MedCalc (20.1.4) to determine the optimized ELISA’s cut-off value [2,3]. Kappa value was determined for ELISA kit by using SPSS. ROC (Receiver Operating Characteristic) curve was also made. This curve is used to realize the accuracy of a newly developed diagnostic test while plotting specificity on X-axis and sensitivity on Y-axis, in the form of "AUC" or area under the curve. The accuracy was estimated as follows:

Accuracy = (True negative + True positive) / (True positive + False positive + True negative + False negative) where True Positive (TP) = No. of samples positive with both the ELISA kits, i.e., with optimized ELISA kit and Commercial ELISA kit; False Positive (FP) = No. of samples positive with the new ELISA kit but negative with the commercial ELISA kit; True Negative (TN) = No. of negative samples with both the kits, i.e., the new ELISA kit and the Commercial ELISA kit; and False Negative (FN) = No. of samples negative with the new ELISA kit but positive with the commercial ELISA kit and Youden Index = (Sensitivity + Specificity) - 1.

### Determination of positive predictive value, sensitivity, negative predictive value, specificity, LR+, LR- and DOR

Positive Predictive Value (PPV), Sensitivity, Negative Predictive Value (NPV), Specificity, Likelihood Ratio for a positive test result (LR+), Likelihood Ratio for a negative test result (LR-) and Diagnostic Odd Ratio (DOR) of optimized ELISA against Commercial ELISA was done through contingency tables. These values were estimated as follows: Sensitivity = {True Positive/(True Positive + False Negative)}*100; Specificity = {True Negative /(True Negative + False Positive)}*100; PPV = {True Positive/(True Positive + False Positive)}*100; NPV = {True Negative/(True Negative + False Negative)}*100; LR+ = Sensitivity/(1-Specificity); LR- = (1-Sensitivity)/Specificity; and Diagnostic Odd Ratio (DOR) = LR+/LR-.

### Ovine seroprevalence through LAT and newly optimized ELISA kit

The 400 sera were evaluated through the protocols of LAT and newly optimized ELISA kit to find out the Ovine Seroprevalence. LAT was done as described by protocol [4], while newly optimized ELISA was performed as mentioned for newly Optimized ELISA kit.

### Evaluation of the comparative efficacy of LAT and Optimized ELISA kit

The cross-sectional study for comparative evaluation of LAT and Optimized ELISA kit was conducted in two regions of the Punjab province: one is tehsil Sharaqpur (an agro-ecological zone), district Sheikhupura, West of Punjab, North-West to Lahore and second is tehsil Kot Chutta (a range-ecological zone), district Dera Ghazi Khan, located in South West of Punjab, Pakistan.

LAT was performed as delineated by protocol [42] (Toxo-Latex, Linear Chemicals, Spain, Latex Kit Lot # 20072712). The slide agglutination at the blackish region indicated complex of antibodies of unknown samples with antigen of *T*. *gondii*. Shortly, each serum sample was diluted to 1:4 with 0.1 M PBS. The 25 μL of diluted serum was mixed on the blackish region of slide. Positive and negative control sera were mixed with the help of spatula. The slide was shaked at a rocker for 3 to 5 min, and the agglutination was observed by gross visualization. The agglutination was considered positive or negative after comparison with the positive and negative controls. Whereas, ELISA was performed as described above for the newly Optimized ELISA kit.

### Evaluation of the comparative efficacy of newly optimized ELISA kit and commercial ELISA kit

Out of 400 sera, 90 were selected on the basis of positive and negative sera through LAT and newly optimized ELISA kit as shown in Table 1.

**Table 1:** The selection of 90 sera.

Selection of sera on the basis of tests (Newly optimized ELISA kit and LAT)	No. of Selected Sera
Newly Optimized ELISA kit^+^ LAT^+^	30
Newly Optimized ELISA kit ^+^ LAT ^-^	20
Newly Optimized ELISA kit—LAT ^-^	20
Newly Optimized ELISA kit—LAT ^+^	20
Total	90

The protocol of commercial ELISA kit was performed as described above for the Optimized ELISA kit. Then the efficacies of both tests (newly optimized ELISA kit and commercial ELISA kit) were evaluated.

### Meteorological data of Sharaqpur and Kot Chutta

The geo-climatic conditions and the sheep population of both areas are shown in Table 2 [1]. This data of Table 2 is for the comparison of population of sheep in both regions and location of regions the both regions in Punjab, Pakistan.

**Table 2:** Coordinates, temperature, rainfall, and sheep population of tehsils Sharaqpur (District Sheikhupura) and Kot Chutta (District DG Khan), Punjab (Pakistan).

Tehsil (District)	Coordinates			Temperatures and rainfall							
				Summer (in July)			Winter (in January)				
	Latitude (°N)	Longitude (°E)	Elevation (from sea level—m)	Max. Temp (°C).	Min. Temp. (°C).	Rainfall (mm)	Max. temp (°C).	Min. Temp. (°C).	Rainfall (mm)	Sheep Population	Breeds of sheep and Population
Sharaqpur	31°27’36.33 "	74°6’7.76 "	**203.911▲**	42	32	**75.45▲**	22	10	**19.72▲**	75353 in Sheikhupura	Lohi = 31024 Kajli = 8551 Thalli = 648
Kot Chutta	29° 53’ 7.6128 "	70°38’ 54.4092"	112	**44▲**	32	27.46	26**▲**	15**▲**	10.41	858437 in D. G. khan	Balochi = 4839 Kajli = 29094 Balkhi = 26267

*Source: Anonymous (2006). [43] ▲: Relatively higher levels of parameters.

For the evaluation of the parameters of meteorological association with ovine toxoplasmosis, the meteorological data of Kot Chutta and Sharaqpur was collected from 2015 to 2020 [44,45].

### Statistical analysis

MedCalc (version 11.4.4.0) was employed to compare the outcomes of the optimized ELISA kit and the Commercial ELISA kit by estimating the kappa value (κ = 0.978). Landis and Koch (1977) provided a reasonable approach to interpreting kappa: very approximately, they regard the agreement as follows: k ≤ 0.2 (Poor), 0.21 ≤ k ≤ 0.40 (Fair), 0.41 ≤ k ≤ 0.60 (Moderate), 0.61 ≤ k ≤ 0.80 (Substantial), k exceeds than 0.80 (Good) [5]. A ROC curve was employed to determine the optimized ELISA’s cut-off value [2,3,46]. The seroprevalence of both the regions (Sharaqpur and Kot Chutta) was analyzed through Chi-square using SPSS. The Odds Ratios (OR), and p values were also calculated through SPSS (IBM SPSS Statistics for Windows, Version 23.0. Armonk, NY: IBM Corp) [6]. The 3D plots were made to find out a direct or indirect correlation between seroprevalence and relative humidity and an inverse or direct correlation between seroprevalence and average temperature. The association of meteorological parameters with seroprevalence of *Toxoplasma* was determined through Partial Coefficient correlation using SPSS Statistics for Windows, Version 23.0 (IBM SPSS Statistics for Windows, Version 23.0Armonk, NY: IBM Corp) and R-4.2.1 software.

## Results

### Optimization of new optimized ELISA kit

For optimization of the new ELISA kit, 90 sheep sera were evaluated by the commercial ELISA kit and the new ELISA kit (the kit to be optimized); the detail of samples found positive and negative in both the kits can be seen in Table 3.

**Table 3:** Sensitivity and specificity of new optimized ELISA kit against commercial ELISA kit.

New optimized ELISA Kit ↓	Commercial ELISA kit		Total
	Positive	Negative	
Positive	49(a)	01(b)	50
Negative	0(c)	40(d)	40
Total	49	41	90

Sensitivity = {a/(a+c)}*100 = {49/49}*100 = 1*100 = 100%.

Specificity = {d/(b+d)}*100 = {40/41}*100 = 0.9756*100 = 97.56%.

(Predicted on equations and formulae reported by Thrushfield [7].

**Fig 1:**
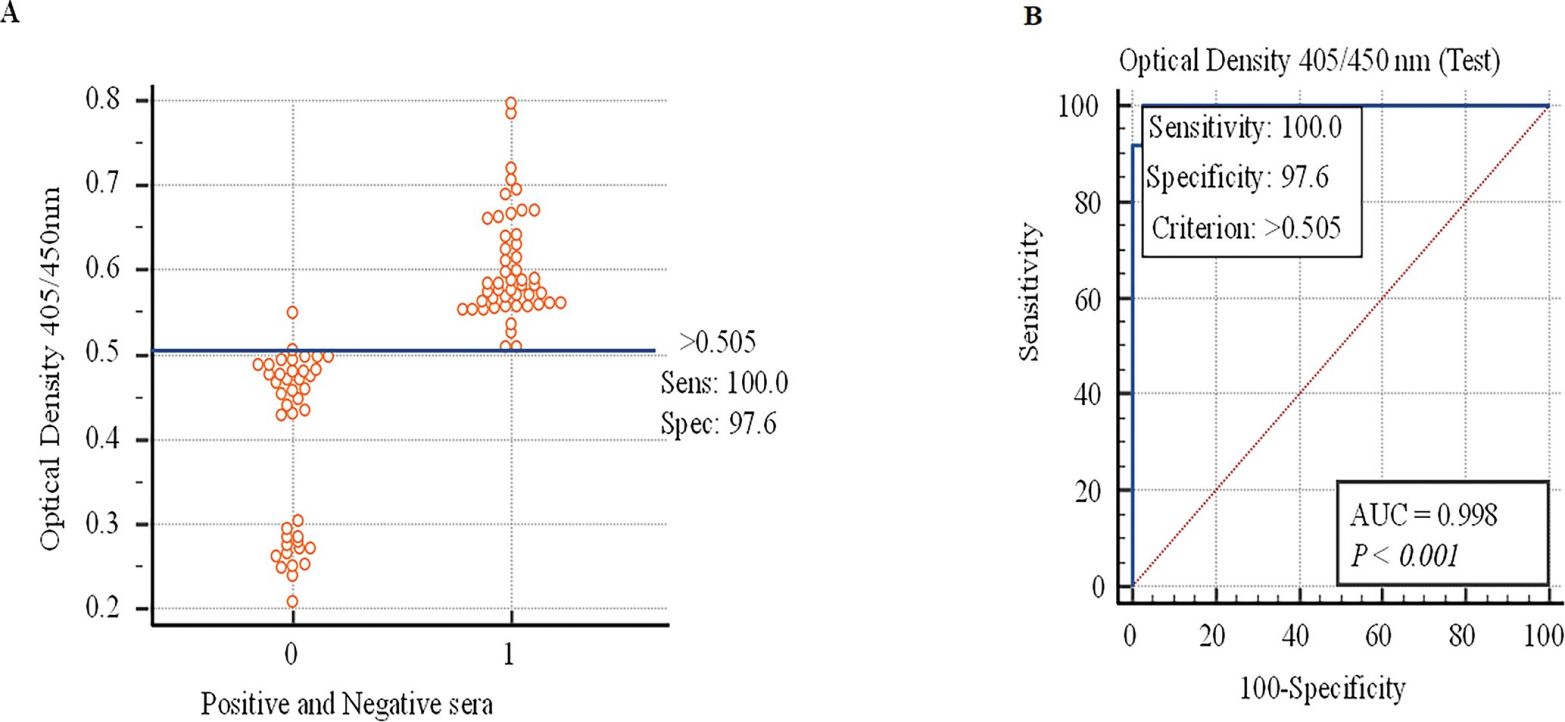
Sensitivity, specificity, and a cut-off value of newly Optimized ELISA kit. (A). ROC curve made using outcomes of Commercial ELISA kit and optimized ELISA kit (B).

100% Sensitivity and 97.6% Specificity of newly Optimized ELISA kit was found through ROC, as shown in Fig 1.

### Seroprevalence of ovine toxoplasmosis through LAT and newly Optimized ELISA kit

Out of 400 sheep samples, 168 (42%) samples were found as seropositive for toxoplasmosis by LAT (Pearson Chi-square (χ2) value 275.156; p < 0.0001) and 133 (33.25%) as seropositive through optimized ELISA (Pearson Chi-square (χ2) value 86.049; p < 0.0001) (Table 4).

**Table 4:** Outcomes of LAT and optimized ELISA for recognition of seroprevalence of ovine toxoplasmosis.

**Outcomes**			**Comparison between LAT and Optimized ELISA kit**			
	Results (LAT)	Results (Optimized ELISA kit)	Odds ratio (95%CI)	Chi-square (χ^2^)	df	P-value
Positive	168 (42%)	133 (33.25%)	1.04 (0.7–1.6)	6.912	01	0.00856
Negativ**e**	232 (58%)	267 (66.75%)				
Total	400	400				

### Seroprevalence of sheep in Kot Chutta

As determined by LAT, the seroprevalence of ovine toxoplasmosis in Kot Chutta was 39.5% (79/200). Seroprevalence of toxoplasmosis in male sheep (15.5%; 31/200) was less as compared to females (24%; 48/200), as described in Table 5.

**Table 5:** Sex-wise distribution of *T*. *gondii* antibodies in sheep of Kot Chutta (D.G. Khan) and Sharaqpur (Sheikhupura) through LAT and newly optimized ELISA kitTehsils.

	**LAT**						**Optimized ELISA Kit**					
	No. of positive		Overall prevalence (%)	Odds ratio (95% CI)	Chi-square (χ^2^)	P-value	No. of positive		Overall prevalence (%)	Odds ratio (95% CI)	Chi-square (χ^2^)	P-value
	Male	Female		0.9 (0.6–1.5)	0.012	0.01	Male	Female		0.9 (0.6–1.5)	0.036	0.001
**Kot Chutta**	31(15.5%)	48 (24%)	79 (39.5%)				24 (12%)	38 (19%)	62 (31%)			
**Sharaqpur**	34 (17%)	55 (27.5%)	89 (44.5%)				27 (13.5%)	44 (22%)	71 (35.5%)			

Looking at different age groups, the highest seroprevalence (22.5%;45/200) was seen in the age group of 1–2 years, followed by 6.5% (13/200) in the age group of 3–4 years, 5.5% (11/200) in the age group of ≤ 1 year, 3.5% (7/200) in the age group 2–3 years and 1.5% (3/200) in the age group ≥ 4 years as described in Table 6.

**Table 6:** Age-wise distribution of *T*. *gondii* antibodies in sheep of Kot Chutta (D.G. Khan) and Sharaqpur (Sheikhupura) through LAT and ELISA kit.

Age groups	LAT					Optimized-ELISA kit				
	No. of positive samples		Sharaqpur and Kot Chutta			No. of positive samples		Sharaqpur and Kot Chutta		
	Kot Chutta	Sharaqpur	Odds ratio (95% CI)	Chi-square (χ^2^)	P-value	Kot Chutta	Sharaqpur	Odds ratio (95% CI)	Chi-square (χ^2^)	P-value
≤1 year	11 (5.5%)	15 (7.5%)	1.0 (0.5–3.5)	0.313	0.001	08 (4%)	18 (9%)	0.9 (0.6–5.4)	1.604	0.001
1–2 years	45 (22.5%)	46 (23%)	1.5 (0.8–2.6)	1.689	0.001	40 (20%)	27 (13.5%)	1.4 (0.4–1.3)	1.710	0.001
2–3 years	07 (3.5%)	13 (6.5%)	1.0 (0.7–5.8)	1.660	0.001	05 (2.5%)	13 (6.5%)	1.0 (0.9–9.6)	3.641	0.001
3–4 years	13 (6.5%)	09 (4.5%)	0.2 (0.2–2.2)	0.235	0.001	08 (4%)	09 (4.5%)	0.6 (0.5–4.8)	0.604	0.001
≥ 4 years	03 (1.5%)	06 (3%)	0.1 (0.1–8.9)	0.000	0.01	01 (0.5%)	04 (2%)	0.3 (0.2–3.5)	0.600	0.01

Seroprevalence of ovine toxoplasmosis of Kot Chutta was detected as 31% (62/200). Seroprevalence of ovine toxoplasmosis in males (12%; 24/200) was less as compared to females (19%;38/200), and it was significant (*p < 0*.*05*).

Looking at different age groups, the highest seroprevalence (20%;40/200) was seen in the age group of 1–2 years, followed by 7.5% (8/200) in the age group of ≤ 1 year, 4% (8/200) in the age group of 3–4 years, 2.5% (5/200) in the age group 2–3 years and 0.5% (1/200) in the age group ≥ 4 years and it was significant (*p < 0*.*001*) (See Tables 5 and 6).

### Seroprevalence of ovine toxoplasmosis in Sharaqpur

Using LAT, the seroprevalence of ovine toxoplasmosis of Sharaqpur was detected as 44.5% (89/200). Seroprevalence of ovine toxoplasmosis in males (17%;34/200) was less as compared to females (27.5%;55/200), and it was significant (*p < 0*.*05*).

Looking at different age groups, the highest seroprevalence (23%;46/200) was seen in the age group of 1–2 years, followed by 7.5% (15/200) in the age group of ≤ 1 year, 6.5% (13/200) in the age group of 2–3 years, 4.5% (9/200) in the age group 3–4 years and 3% (6/200) in the age group ≥ 4 years and it was significant (*p < 0*.*05*).

Using an optimized ELISA kit, the seroprevalence of ovine toxoplasmosis of Sharaqpur was detected as 35.5% (71/200). Seroprevalence of toxoplasmosis in male sheep (13.5%;27/200) was less as compared to females (22%;44/200), and it was significant (*p < 0*.*001*).

Looking at different age groups, the highest seroprevalence (13.5%;27/200) was seen in the age group of 1–2 years, followed by 9% (18/200) in the age group of ≤ 1 year, 6.5% (13/200) in the age group of 2–3 years, 4.5% (9/200) in the age group 3–4 years and 2% (4/200) in the age group ≥ 4 years and it was significant (*p < 0*.*0001*) (See Tables 5 and 6).

### Comparative Efficacy of LAT and Optimized ELISA for ovine toxoplasmosis

The efficacies of both tests (LAT and newly Optimized ELISA kit) have been described in Table 7.

**Table 7:** Efficacy of LAT for recognition of *Toxoplasma*-specific IgG antibodies in 400 sheep sera.

Outcomes	Recombinant Surface Antigen 1 (%)
Sensitivity	79.16
Specificity	86.89
Positive predictive value	79.16
Positive predictive value	86.89
Validity	83.025
Kappa value (95% CI)	0.815 (0.000–0.000)
Standard error	0.029
Accuracy	85.96
χ2 = 275.156	
*P < 0*.*0001*	

### Characterization of newly Optimized ELISA kit

The cut-off (point) value determined using statistical software (MedCalc) was 0.505. This value for optimized ELISA was employed to decide the positive and negative sheep sera that were evaluated by the optimized ELISA kit. Kappa value came out to be 0.978 and Youden Index = 196.6, a highly desirable value (*P< 0*.*0001*) connecting optimized ELISA and Commercial ELISA as delineated in Tables 8 and 9, thus showing high confidence in our new optimized ELISA kit.

**Table 8:** Characterization of optimized ELISA kit based on commercial ELISA kit for detecting specific IgG antibodies against *T*. *gondii* in 90 sera.

Outcomes	Recombinant Surface Antigen 1 (%)
Sensitivity	100
Specificity	97.56
Positive Predictive Value (PPV)	98
Negative Predictive Value (NPV)	100
Validity	98.78
Relative agreement	90.816
Kappa value (95% CI)	0.978 (0.956–1.000)
Standard error	0.00221
Area Under Curve (AUC)	0.998
Accuracy	98.88
χ2 = 86.049	
*P* < 0.0001	

**Table 9:** LR+, LR-, and DOR of optimized ELISA kit.

Optimized ELISA ↓	Commercial ELISA		Total
	Positive	Negative	
Positive	49.5(a)	1.5(b)	51
Negative	0.5(c)	40.5(d)	41
Total	50	42	92

LR+ for positive sera = {a/(a+c)} / {b/(b+d)} = {49.5/50} / {01.5/42} = 28.28.

LR- for negative sera = {c/(a+c)} / {d/(b+d)} = {0.5/50} / {40.5/42} = 0.0104.

DOR = LR+/LR- = 28.28/0.0104 = 2719.23.

{Predicted based on the formulae and table of [47]}.

For optimized ELISA, AUC was 0.998, thus displaying its high accuracy as narrated earlier [8]. The test is considered highly accurate if a test has an AUC equal to or more than 0.9.

### Meteorological parameters and seroprevalence of Sharaqpur and Kot Chutta

**Fig 2:**
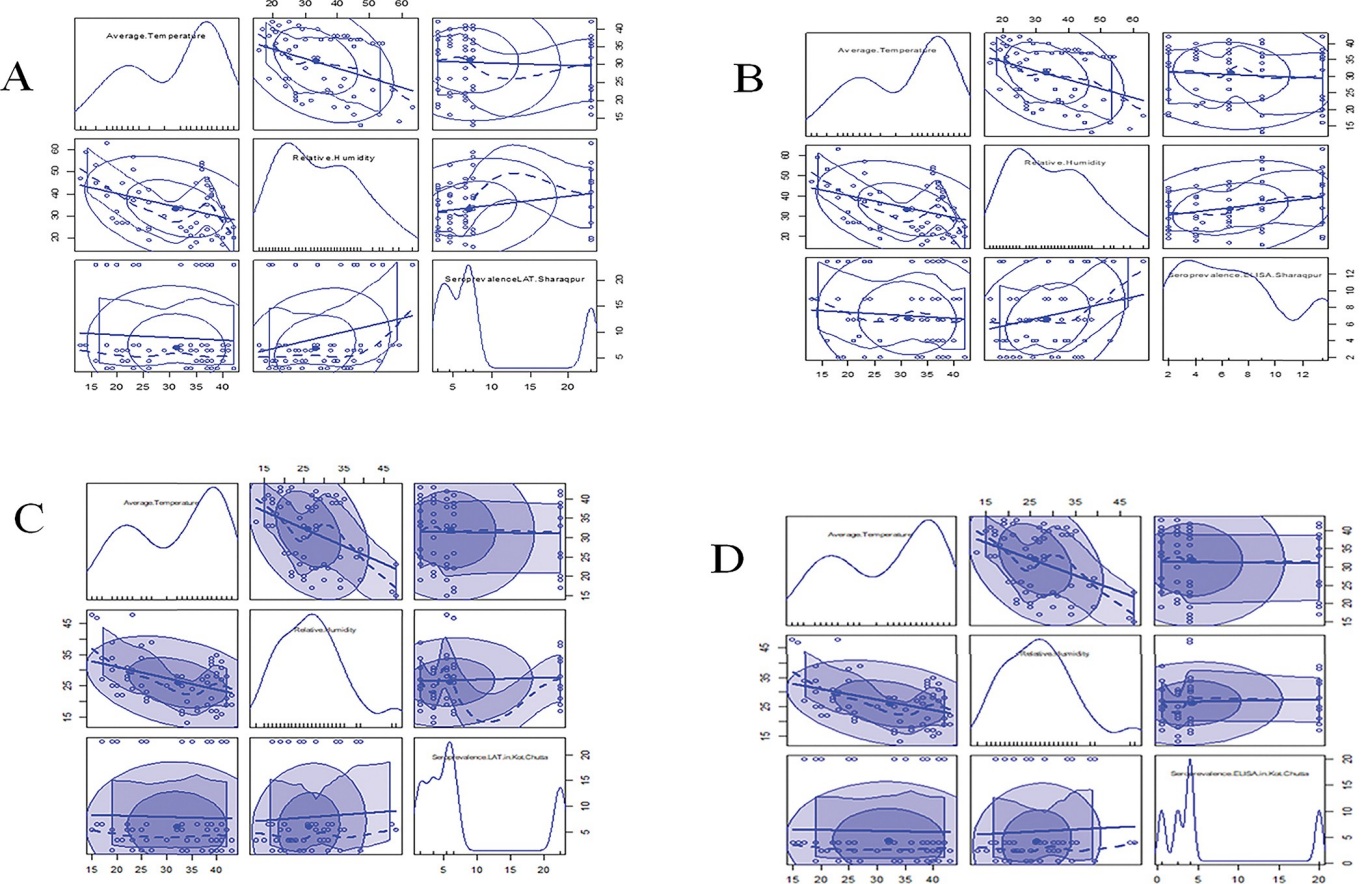
Correlation of meteorological data of Sharaqpur with seroprevalence through LAT and ELISA (A, B) and meteorological data of Kot Chutta with seroprevalence through LAT and ELISA (C, D) during the years 2015–2020.

The average summer and winter rainfall in Sharaqpur were higher than that of Kot Chutta, whereas the average temperature range of Sharaqpur was lower than that of Kot Chutta in the summer and winter seasons. During the years (2015–2020), the range of monthly average temperature (38-40°C) of Sharaqpur was found to be lower relative to that (41-43°C) of Kot Chutta. On the other hand, the range of monthly average relative humidity (27–50%) in May, June, July, and August was higher relative to that (22–35%) of Kot Chutta for the same months (Fig 2).

The seroprevalence of ovine toxoplasmosis was found more in Sharaqpur than in Kot Chutta. The correlation of meteorological parameters (Minimum temperature, Maximum temperature, Average temperature, and Relative humidity) and ovine seroprevalence of both zones (agro-ecological and range-ecological) through LAT and optimized ELISA kit depicted that relative humidity had positive effects. As relative humidity increased, the level of ovine toxoplasmosis also increased in Sharaqpur, thus indicating a potentially higher survival of the parasite in the environment. While in Kot Chutta, relative humidity was found less, and temperatures were high compared to Sharaqpur. So, the high temperature was found to be negatively correlated with the seroprevalence of ovine toxoplasmosis, thus indicating an adverse impact of environmental temperature on the survival of *T*. *gondii* oocysts in the environment. Similarly, relative humidity was positively correlated with seroprevalence of ovine toxoplasmosis as lower seroprevalence of ovine toxoplasmosis was recorded in Kot Chutta with lower relative humidity, as shown in Table 10.

**Table 10:** Correlation of meteorological parameters and seroprevalence of both zones.

Parameters	Partial coefficient correlation with seroprevalence			
	Zones	Tests	r_partial_	p
T_max_	Agro-ecological	LAT	0.991	0.000
T_avg_			0.975	0.000
T_mini_			0.966	0.000
RH_avg_			-0.320	0.013
T_max_		Optimized ELISA	0.991	0.000
T_avg_			0.969	0.000
T_mini_			0.978	0.000
RH_avg_			-0.325	0.012
T_max_	Range-ecological	LAT	0.981	0.000
T_avg_			0.973	0.000
T_mini_			0.974	0.000
RH_avg_			-0.325	0.012
T_max_		Optimized ELISA	0.981	0.000
T_avg_			0.973	0.000
T_mini_			0.974	0.000
RH_avg_			-0.325	0.012

### Meteorological parameters and seroprevalence of different age groups of sheep

**Fig 3:**
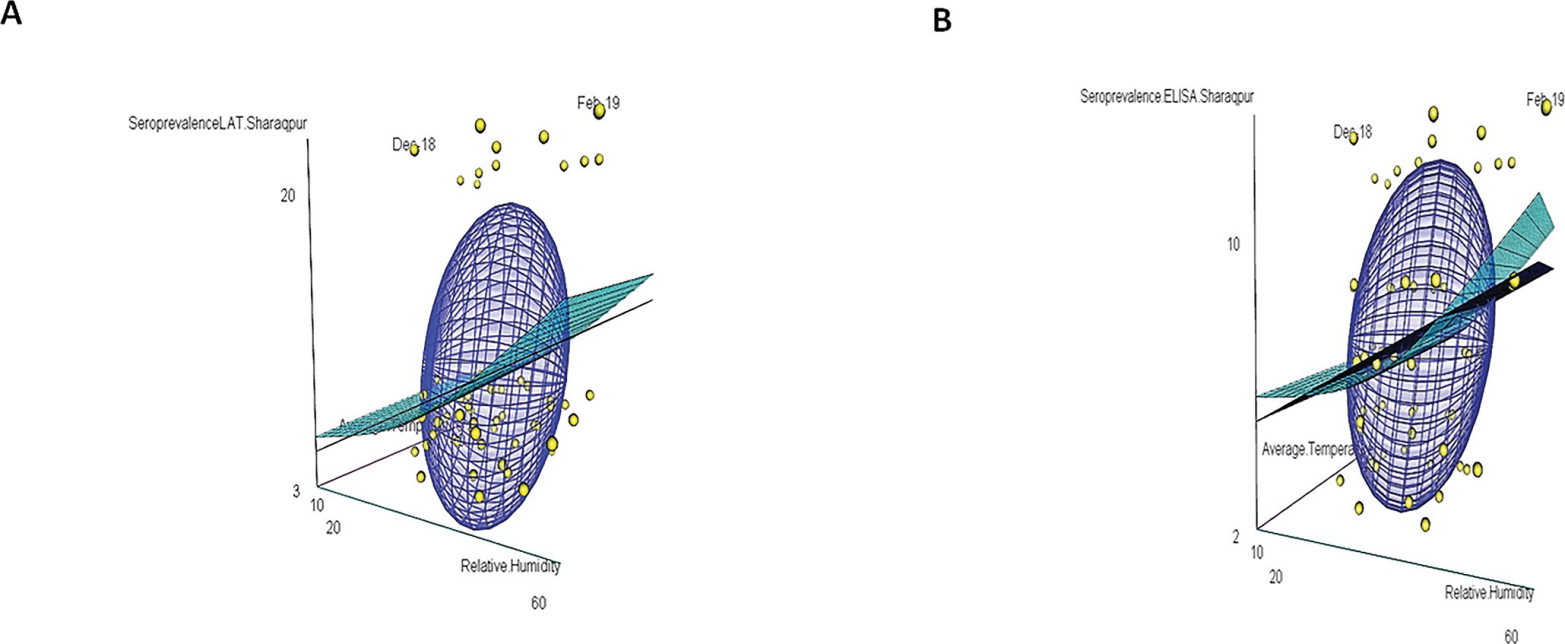
Meteorological parameters (average monthly temperatures and average monthly relative humidity grouped into yearly units relative to age groups (birth years) of Sharaqpur during the year 2015–2020 have a significant correlation with the age-wise seroprevalence of ovine toxoplasmosis determined both through LAT (A) and ELISA (B), displayed as 3D plots.

**Fig 4:**
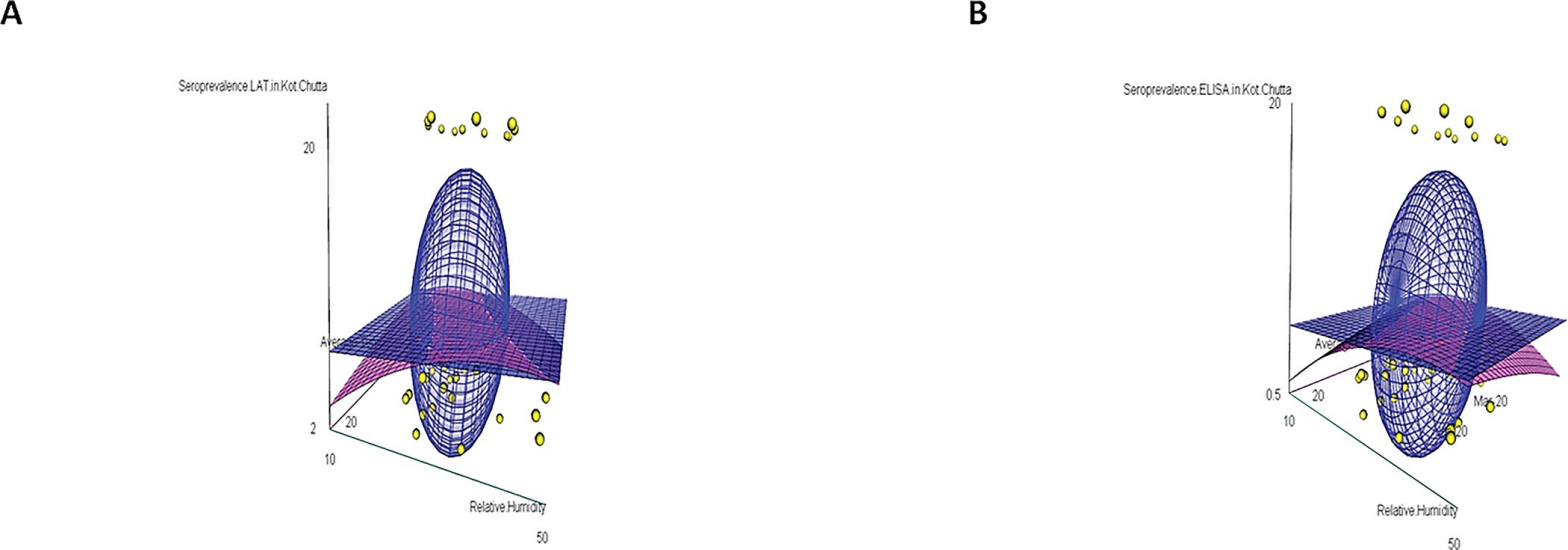
Meteorological parameters (average monthly temperatures and average monthly relative humidity grouped into yearly units relative to age groups (birth years) of Kot Chutta during the year 2015–2020 have a significant correlation with the age-wise seroprevalence of ovine toxoplasmosis determined both through LAT (A) and ELISA (B), displayed as 3D plots.

The ovine toxoplasmosis in the age group 1–2 years [the age-group of 1–2 years (December 2018- November 2019) in Sharaqpur and the age group of 1–2 years (August 2018- July 2019) in Kot Chutta] was found significantly higher as compared to other age groups (≤ 1 year, 2–3 years, 3–4 years and ≥ 4 years) in both the zones (agro-ecological and range-ecological). The meteorological parameters plotted in Fig 2 displays the correlation between age-group-wise-seroprevalence, yearly units of relative humidity, and temperatures. These plots indicate a direct correlation between seroprevalence and relative humidity and an inverse correlation between seroprevalence and average temperature, as shown in Figs 3 and 4.

## Discussion

The Area of Under Curve (AUC) and a cut-off value of optimized ELISA were calculated by ROC as was determined in a previous study [48]. In the current study, optimized ELISA was validated, the cut-off value was 0.505, and the AUC for optimized ELISA was 0.978 (P < 0.001), indicating that this optimized ELISA is a highly accurate test kit.

The new optimized ELISA kit was developed using a recombinant form of Toxoplasma’s surface antigen-1 (rSAG1). The methodology was the same as reported earlier, with minor modifications (46). Thus, the rSAG1 in the optimized ELISA kit was employed @ 0.125μg/mL for coating of ELISA plate. The high accuracy at this low concentration is an advantage in terms of the economical use of this recombinant protein for coating ELISA plates. The optimum dilution of sheep serum for use in an optimized ELISA kit was 1/1600. This value was determined by studying all the 90 samples used for optimization. Similarly, the optimum duration for reading OD values after the addition of substrate was determined as 05 min based on data obtained from comparing OD values of 90 samples, read at various time intervals, i.e., 05 min., 10 min., 15 min., and 20 min. Subsequently, a secondary antibody was found to show optimum effect at a dilution of 1/10,000, lower than the dilution used previously for developing an ELISA kit of human toxoplasmosis [42]. The secondary antibody incubation duration was found optimum as two hours- the same duration as previously used [42]. 37°C was found as an optimum temperature for the incubation, as determined earlier [42]. These optimized conditions of protein concentrations, serum and conjugate dilutions, incubation temperature, and optimized time (OPT) for reading OD, showed good diagnostic values of our newly optimized sheep ELISA kit.

Commercial ELISA is a benchmark and is a confessed accurate serodiagnostic method for conducting Toxoplasma surveillance in various animals like sheep, goats, and bovines [16]. The commercial kit was employed to assess the serological status of sheep to detect toxoplasmosis. In a study, Mangili et al. (2009) used an ELISA kit (ID Screen Toxoplasmosis Indirect Multi-species) as a pennant for the development of the procedure of ELISA for the identification of Toxoplasma-specific antibodies in Sheep [31]. The Gold Standard ELISA kit (Commercial ELISA kit) employed in the current study has a protocol of only 90 min and having the 82.48% sensitivity and 97.8% specificity.

LAT and optimized ELISA kits revealed 42% (168/400) and 33.25% (133/400) samples as seropositive for toxoplasmosis, respectively, with significantly higher seropositivity in the Sharaqpur as compared to Kot Chutta. The seroprevalence determined through the optimized ELISA kit was lower than that observed by LAT. This difference can be expected as there can be several factors behind this difference. LAT is known to produce false positive results [49–51]. In addition, the variation may be due to the breed difference as the animals sampled were the mixed local breed of sheep that may be more prone to toxoplasmosis. Similar levels of seroprevalence have been seen in sheep in Charsadda (40%), a district located in North Pakistan [52] using LAT, and in Mardan (44.13%) using Indirect Hemagglutination Antibody (IHA) assay [53]. The prevalence of *T*. *gondii* in sheep and goats reported from the various regions of Iran also falls in the close range, i.e., 24.5% and 33.3%, respectively [54,55].

The humid and temperate climate is considered one of the most common and important factors in the high prevalence of *T*. *gondii*. Sheep constitute the common source of meat production in Kot Chutta (Dera Ghazi Khan) and Sharaqpur (Sheikhupura), as in the rest of Pakistan. The consumption of undercooked or raw mutton sheep is one of the major risk factors for toxoplasmosis [56]. Kot Chutta and Sharaqpur are geographically distant regions in Punjab, Pakistan. This is the first work of seroprevalence of ovine toxoplasmosis in Sharaqpur (from district Sheikhupura) and Kot Chutta (from district D.G. Khan), Punjab (Pakistan). In this cross-sectional study, a total of 400 sheep sera (Kot Chutta: *n* = 200; Sharaqpur: *n* = 200), were scanned serologically for *T*. *gondii*-specific antibodies. Significantly higher seroprevalence (44.5% by agglutination; 35.5% by ELISA) was detected in Sharaqpur as against that (39.5% by agglutination; 31% by ELISA) in Kot Chutta (P < 0.01; χ2). Analysis of meteorological data from both the regions points towards the high rainfall, low environmental temperatures, and high altitude as the important factors behind the significant difference in seroprevalence level, and thus potentially behind the enhanced risk of toxoplasmosis to grazing sheep, owing to a more favorable coccidian oocyst-survival in Sharaqpur. Sharaqpur is a greenish area located at a higher latitude (31°27’36.33°N), at a higher elevation from sea level (203.911m), with higher average rainfall (19.72mm) as compared to Kot Chutta, is a plain area, at a lower latitude (29°53’7.6128°N), at a lower elevation from sea level (112m), with lower average rainfall (10.41mm).

The seroprevalence of *T*. *gondii* was found relatively less in males as compared to female animals- and this is true in the case of detection through both techniques, i.e., LAT and optimized ELISA kit, as well as for both the regions. Higher susceptibility of female animals to protozoan parasites as compared to male ones has already been reported in the literature [5,57–59]. It has also been reported in the literature that males are less susceptible to toxoplasmosis [56,60].

Another interesting finding of the current study is that the highest seroprevalence was found in the animals having an age of 1–2 years whereas the lowest seroprevalence was seen in the oldest animals i.e., the animals aged more than 4 years. This was true for both the geographical regions as well as through both the diagnostic techniques compared in the study. This high rate of prevalence in animals aging 1–2 years points towards the higher susceptibility of this particular age group of sheep to toxoplasmosis on one hand whereas it may also indicate the availability of more favourable environmental factors in the birth year of this age-group as well as in the years following their birth year. For this aspect, there is a need to further explore the meteorological data to find out more specific explanation for this phenomenon as this particular age group in sheep, makes the significant part of animals meant for mutton production.

LAT revealed a higher seroprevalence of toxoplasmosis in sheep samples from both the regions tested (9% higher in Sharaqpur; 8.5% higher in Kot Chutta) as compared to the values detected through optimized ELISA (Sharaqpur: LAT: 44.5%; ELISA: 35.5%; Kot Chutta: LAT: 39.5%; ELISA: 31%).

One crucial point is that through LAT, the highest seroprevalence was found in the age group 1–2 years, whereas 3.5 times less seroprevalence was seen in the age group 3–4 years in Kot Chutta. Normally, we consider that the seroprevalence of toxoplasmosis in all age groups of sheep are alike without involvement of environmental factors. As the cur-rent study assumed the involvement of environmental factors having significant impact, the difference of seroprevalence found among the various age groups of sheep, could be because of this effect. Subsequently, among these different age groups, 1–2 years age group was found more prone to get the infection of toxoplasmosis probably due to conducive environment for parasite-survival in their birth year.

LAT is used as an initial screening test for identification of toxoplasmosis, having relatively less sensitivity and specificity [61] as compared to the optimized ELISA, with high sensitivity (100%) and specificity (97.6%). LAT is an agglutination test where Toxoplasma antigen can react with specific antibodies whatsoever it may be IgM or IgG, whereas the ELISA kit was specifically designed for detection of only Toxoplasma-specific IgG. Therefore, this fact may be one of the reasons behind the apparent difference in sero-prevalence values detected through LAT and ELISA. Because of this reason, we can expect to have higher seroprevalence while using LAT as compared to the ELISA. LAT has sensitivity of 79.1% and specificity of 86.89% as described by the manufacturer. While in the current study, the values of sensitivity and specificity of newly optimized ELISA kit (Sheep Toxo IgG ELISA Kit) surpassed those values for LAT. False positivity of LAT is already described [62], as indicated through the values of optimized ELISA kit. LAT is not considered a reliable and perfect method to identify the *T*. *gondii* infection [48].

LAT is not considered a substantative test regarding the diagnosis of toxoplasmosis [50,63]. The optimized ELISA kit is used for identification of only IgG antibodies while LAT is not specific for IgG antibodies. So therefore, Immunoglobulin M (IgM) antibodies of the various sheep sera may react non-specifically to Toxoplasma whole antigen used in LAT in a similar way as found in some other host species undergoing Toxoplasma-seroprevalence and depicting false positivity. The non-specific interaction of IgM with Toxoplasma-antigen and of Bovine Serum Albumin (BSA) of sera with latex [64]—due to a mimicry existing between conserved PAMPs (Pathogen-associated molecular patterns) across species—are likely reasons behind false positivity of LAT and advocate for further investigation. Moreover, another likely reason behind this apparent difference in the sero-prevalence of ovine toxoplasmosis detected by LAT (44.5%) and optimized ELISA kit (35.5%) might be the fact that both types of antibodies i.e. IgM and IgG can interact with Toxoplasma total antigenic extract used in the LAT as against the sole detection of IgG in the newly optimized ELISA kit using a single recombinant antigen. The same reasons are the likely contributing factors behind the reduced rate of seroprevalence detected in ELISA kit as against that found through LAT.

It is known that toxoplasmosis in animals can occur by eating infective oocysts from the environment [59,65]; hence the likelihood of getting infected from the environment is always there. While the animal ages, its cumulative likelihood of getting infected increases. That is why the age of animals is considered one of the most crucial factors in finding the prevalence rate of toxoplasmosis in animals [66]. In contrast to this understanding, the age group 1–2 years comes out with the highest seropositivity (45%) through LAT and optimized ELISA kit from both the ecological zones in our study. In our findings, the sero-positivity of *T*. *gondii* infection was higher in Sharaqpur (Sheikhupura) than in Kot Chutta (D.G. Khan). In a study, Bastiaan G. et al. (2009) described that environmental factors usually affect the prevalence of toxoplasmosis. Three different effects on *T*. *gondii* were identified: (1) the survival of the pathogen affected by environmental changes; (2) due to in-creased rain or snow, sporulated oocysts dispersed throughout the environment; and (3) climate change influenced the ecology of hosts (transport). Environmental conditions are essential for the survival of oocysts. Worldwide, the prevalence of toxoplasmosis is high in tropical and humid areas and low in dry and hot areas [67]. According to the Livestock Census of Punjab (Pakistan) (2006) and the world weather system, the level of rainfall in the Winter and Summer seasons is high in Sharaqpur as compared to Kot Chutta [68]. That may be another reason for a higher seroprevalence of ovine toxoplasmosis seen in Sharaqpur compared to Kot Chutta.

In the extensive grazing system of sheep, the presence of wild and domestic felines spreading oocysts of *T*. *gondii* contaminates the environment in the more greenish area like Sharaqpur, enhancing the opportunity of sheep getting infected during grazing. Similar findings have been seen in Guilan province, Iran, in a study performed by Chaechi Nosrati et al. (2020) and in Nepal by Subedi et al. on sheep [48,56]. Such oocysts can survive in humid and moderate climates for up to 18 months [50]. In the current study, high rain-fall, low temperature, and high elevation seem to contribute to oocyst survival in the environment. This can lead to a higher possibility of Toxoplasma infection in grazing sheep because of enhanced exposure to a higher percentage of Toxoplasma oocysts survived from cat feces in Sharaqpur compared to Kot Chutta.

In this study, no connection was found between health status and seropositivity of toxoplasmosis in sheep. All the seropositive animals displayed satisfactory health status and looked normal.

A newly optimized ELISA kit is highly effective in determining the seroprevalence of Toxoplasma in sheep. A high prevalence of ovine toxoplasmosis in Sharaqpur observed in this study appears to be related to the region’s high rainfall, high elevation, and high humidity. A novel significant correlation was found between the meteorological parameters (relative humidity, minimum, maximum, and average temperatures) divided into yearly units of both the ecological zones with year-wise seroprevalence (based on the birth years of age-wise groups) of the corresponding regions. Due to favorable climatic conditions, the environmental survival of Toxoplasma is assumed to be higher in the Sharaqpur region than in Kot Chutta region. The higher seroprevalence of ovine toxoplasmosis in sheep of Sharaqpur, as compared to that in Kot Chutta, strengthens this hypothesis.

## Conclusions

Newly ELISA kit was optimized through commercial ELISA kit by the evaluation of 90 sheep sera. Newly optimized ELISA kit (named as Sheep Toxo IgG ELISA kit) has 100% sensitivity, 97.6% specificity, 98% PPV, 100% NPV, 28.28 LR+, 0.0104 LR-, and 2719.23 DOR. Seroprevalence of ovine toxoplasmosis was detected higher in agro-ecological zone (44.5% by LAT; 35.5% by ELISA) than range-ecological zone (39.5% by LAT; 31% by ELISA). Highest seroprevalence was seen in age group of 1–2 years and the lowest in (≥ 4 years). As compared to the efficacies of LAT and commercial ELISA kit, newly optimized ELISA kit showed better results. Newly optimized ELISA kit (named as Sheep Toxo IgG ELISA kit) has 100% sensitivity, 97.6% specificity, 98% PPV, 100% NPV, 28.28 LR+, 0.0104 LR-, and 2719.23 DOR. Sheep Toxo IgG ELISA kit has been evidenced in the current study as more reliable and well-founded immunodiagnostic kit as compared to LAT. The seroprevalence of ovine toxoplasmosis in Sharaqpur and Kot Chutta was found having a significant relationship with the meteorological parameters (positive correlation with relative humidity and negative correlation with temperature). Further studies should be done in other parts of the world involving other ecological zones to ratify the relationship of ovine toxoplasmosis to meteorological parameters.

## Supporting information

S1 File Contains all the supporting files. (ZIP)

## Abbreviations

CI: Confidence interval
T. gondii: *Toxoplasma gondii*
OR: Odds ratio
